# Investigation of cell wall proteins of *C. sinensis* leaves by combining cell wall proteomics and N-glycoproteomics

**DOI:** 10.1186/s12870-021-03166-4

**Published:** 2021-08-20

**Authors:** Yanli Liu, Linlong Ma, Dan Cao, Ziming Gong, Jing Fan, Hongju Hu, Xiaofang Jin

**Affiliations:** Fruit and Tea Research Institute, Hubei Academy of Agricultural Sciences, No. 10 Nanhu Road, Wuhan, 430064 Hubei People’s Republic of China

**Keywords:** *C. sinensis*, Cell wall proteomics, N-glycoproteomics, Glycoside hydrolases

## Abstract

**Background:**

*C. sinensis* is an important economic crop with fluoride over-accumulation in its leaves, which poses a serious threat to human health due to its leaf consumption as tea. Recently, our study has indicated that cell wall proteins (CWPs) probably play a vital role in fluoride accumulation/detoxification in *C. sinensis*. However, there has been a lack in CWP identification and characterization up to now. This study is aimed to characterize cell wall proteome of *C. sinensis* leaves and to develop more CWPs related to stress response. A strategy of combined cell wall proteomics and N-glycoproteomics was employed to investigate CWPs. CWPs were extracted by sequential salt buffers, while N-glycoproteins were enriched by hydrophilic interaction chromatography method using *C. sinensis* leaves as a material*.* Afterwards all the proteins were subjected to UPLC-MS/MS analysis.

**Results:**

A total of 501 CWPs and 195 CWPs were identified respectively by cell wall proteomics and N-glycoproteomics profiling with 118 CWPs in common. Notably, N-glycoproteomics is a feasible method for CWP identification, and it can enhance CWP coverage. Among identified CWPs, proteins acting on cell wall polysaccharides constitute the largest functional class, most of which might be involved in cell wall structure remodeling. The second largest functional class mainly encompass various proteases related to CWP turnover and maturation. Oxidoreductases represent the third largest functional class, most of which (especially Class III peroxidases) participate in defense response. As expected, identified CWPs are mainly related to plant cell wall formation and defense response.

**Conclusion:**

This was the first large-scale investigation of CWPs in *C. sinensis* through cell wall proteomics and N-glycoproteomics. Our results not only provide a database for further research on CWPs, but also an insight into cell wall formation and defense response in *C. sinensis*.

**Supplementary Information:**

The online version contains supplementary material available at 10.1186/s12870-021-03166-4.

## Background

Plant cell walls are a primary subcellular structure and are located in the outside of the cells. They offer the skeletal framework to tissues and play essential roles in protection, cell-to-cell adhesion and communication. Cell walls are mainly composed of complex polysaccharidic networks of celluloses, hemicelluloses, and pectins with a small proportion of cell wall proteins (CWPs), lignins, and lipids [1]. Among them, CWPs constitute around 10% of cell wall dry weight [2–4], but play important roles in various kinds of biological processes including cell wall metabolism, cell wall composition and modification, cell enlargement, signal transduction, biotic and abiotic stress response and other physiological processes [5–10].

In view of the importance of CWP function, the identification and characterization of CWPs have been widely studied in some plant species such as Arabidopsis [11–19], *B. distachyon* [20–22], flax [23, 24], sugarcane [10, 25, 26], rice [27–29], and others in recent decades by cell wall proteomics strategy using destructive and non-destructive extraction methods. These studies have greatly contributed to a broader knowledge of CWPs. However, to our knowledge, there is still a lack of understanding about CWPs due to difficult extraction and high contamination of intracellular proteins. N-Glycosylation is a common form of eukaryotic protein post-translational modification, and most plant proteins are N-glycosylated through the conventional endoplasmic reticulum (ER)-golgi apparatus (GA) secretory pathway [30, 31]. Consequently, N-glycosylation of plant CWPs is particularly prevalent and extensive. Conversely, large-scale and detailed characterization of N-glycoproteins has a great potential to increase our understanding of CWPs, and therefore N-glycoproteomics can be employed to investigate CWPs [32–36]. Based on these findings, this study combined N-glycoproteomics and cell wall proteomics to investigate CWPs.

*C. sinensis* is an important woody economic crop cultivated widely from tropical to temperate regions, its leaves are usually used for making tea. It is reported that the leaves of *C. sinensis* can accumulate much higher level of fluoride (F) than those of most other plants without exhibiting any toxicity symptoms under normal soil conditions [37–40], suggesting that there may be a special mechanism responsible for F accumulation/detoxification. Previous research has shown that cell wall immobilization and vacuolar compartmentation contribute to F accumulation/detoxification [40, 41], and recently we have found the important roles of CWPs in F accumulation/detoxification by a comparative proteomics analysis [42]. However, CWP identification and characterization have rarely been studied in *C. sinensis* up to now.

Herein, to broaden the knowledge of CWPs and provide a base for revealing the molecular mechanisms underlying F accumulation/detoxification-related CWPs, cell wall proteomics and N-glycoproteomics profiling of *C. sinensis* leaves was performed. In this study, CaCl_2_, EGTA, and LiCl were used sequentially to extract CWPs, and hydrophilic interaction chromatography (HILIC) was also employed to enrich N-glycoproteins. The peptides of obtained proteins were analyzed by ultrahigh performance liquid chromatography coupled with tandem mass spectrometry (UPLC-MS/MS). Afterwards, all the identified proteins were subjected to multiple bioinformatics analysis. All in all, 578 CWPs were identified by combining cell wall proteomics and N-glycoproteomics. This study makes first attempt to investigate cell wall proteome and N-glycoproteome in *C. sinensis.* Our results will expand the understanding of CWPs and reveal the mechanism related to plant growth, development, and defense response.

## Results

### Identification of CWPs

**Fig. 1:**
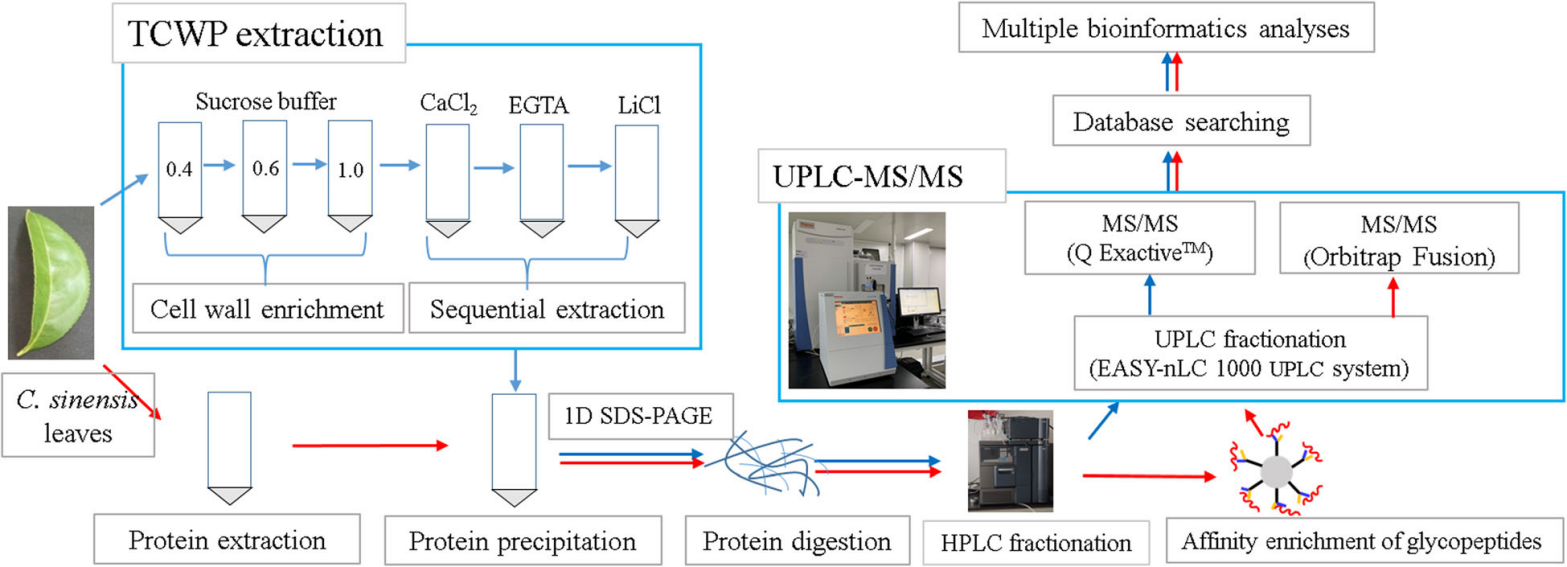
Experiment workflow. The extraction, precipitation, digestion, fractionation, and MS/MS and data analyses of TCWPs were operated according to blue arrow instruction. Likewise, those of glycoproteins were operated according to red arrow instruction. The figure is under copyright, each researcher can use and adapt it by citing our paper

To identify more CWPs, a combining strategy of cell wall proteomics and glycoproteomics was employed in this study as shown in Fig. 1. After UPLC-MS/MS analysis and database search, a total of 3618 target CWPs (TCWPs) and 262 N-glycoproteins were identified from *C. sinensis* leaves (Additional files 1, 2: Table S1, S2). To pick out CWPs, all identified proteins (3880) were subjected firstly to WallProtDB database retrieval. Among them, 627 TCWPs and 187 N-glycoproteins were identified as potential CWPs.

**Fig. 2:**
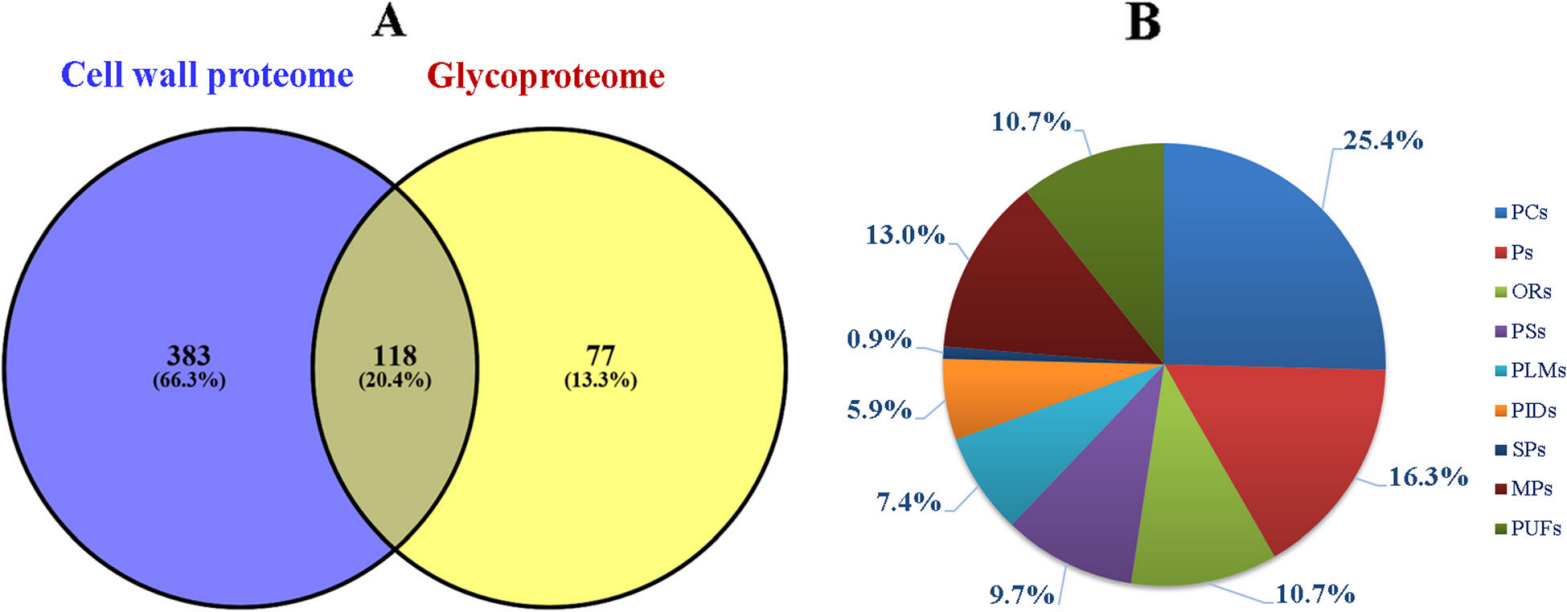
Identification (A) and functional classification (B) of CWPs identified from *C. sinensis* leaves

According to previous reports, only those proteins (i) having a predicted signal peptide (SP), (ii) lacking ER retention signal (KDEL or HDEL) and (iii) no more than one transmembrane domain (TMD) were defined as CWPs [11, 12, 25]. To obtain CWPs as many as possible and enhance CWP coverage, all identified proteins were subjected to multiple bioinformatics analyses including SP, TMD, ER retention signal, and subcellular localization. Based on the above-mentioned definition of CWPs and the report by Day et al. [24], a total of 501 TCWPs and 195 N-glycoproteins were identified as CWPs. Among them, 484 TCWPs and 187 N-glycoproteins were also retrieved in WallProtDB database, whereas 17 TCWPs and 8 N-glycoproteins were absent and thus determined firstly as CWPs (Table 1; Additional file 3: Table S3). As for the remaining proteins, 38 TCWPs were designated as plasma membrane proteins (Additional file 4: Table S4), others including 3079 TCWPs and 67 N-glycoproteins was defined as intracellular proteins. Taken together, 501 CWPs were identified by cell wall proteomics and 195 CWPs were identified by N-glycoproteomics, respectively, 118 CWPs were in common through both approaches (Table 1; Fig. 2A; Additional file 3: Table S3).

**Table 1:** 578 CWPs identified from *C. sinensis* leaves

	Cell wall proteome	Glycoproteome	In common
***Number of identified CWPs***	**501**	**195**	**118**
***Proteins acting on cell wall polysaccharides***	**132**	**43**	**28**
Glycoside hydrolases (GHs)	97	36	23
Carbohydrate esterase family 8 (CE8)	14	2	2
Glycosyl transferases (GTs)	4	0	0
Expansins	6	0	0
PNGase A	2	3	1
Pectin acetylesterases (PAEs)	3	1	1
Pectate lyases (PLs)	2	0	0
homologous to *A. thaliana* PMR5 (Powdery Mildew Resistant) (carbohydrate acylation)	4	1	1
***Proteins involved in signaling***	**41**	**30**	**15**
Leucine-rich repeat receptor-like protein kinases (LRR-RLKs)	14	10	3
Receptor-kinases (RLKs, Gnk-2 homologous domain)	5	4	3
S-locus receptor kinases (SD-1)	2	1	0
Lectin receptor kinases (malectin domain)	2	5	1
Wall-associated receptor kinases (WAKLs)	2	2	1
Fasciclin-like arabinogalactan proteins (FLAs)	8	6	5
Expressed protein (LRR domains)	4	0	0
Expressed protein	2	2	2
Homologous to rapid alkalinization factor (RALF)	2	0	0
***Proteases***	**85**	**34**	**25**
Serine carboxypeptidase S10	22	8	7
Serine carboxypeptidase S28	5	2	2
Asp protease(Peptidase family A1)	23	10	5
Cys proteases(Peptidase family C1) (Papain family)	13	4	3
Ser protease (Peptidase family S8)(Subtilisin)	18	7	6
Subfamily M20A unassigned peptidases	1	2	1
Peptidase M28	1	1	1
Peptidase C13 (legumain family)	1	0	0
DUF239	1	0	0
***Proteins with interaction domains (with proteins or polysaccharides)***	**31**	**7**	**4**
Plant invertase/pectin methylesterase inhibitors (PMEI)	3	1	0
Proteinase inhibitor family I25 (cystatin family)	5	0	0
Expressed proteins (X8 domain)	3	1	1
PGIPs	2	0	0
Kunitz-P family	3	0	0
Expressed proteins (LRR domain)	5	1	1
Lectin receptor kinases (legume lectin domain)	2	2	1
Serpin (Serine protease inhibitor)	1	0	0
Trypsin and protease inhibitor	1	0	0
lysM domain	1	1	0
Ribosome inactivating protein	5	1	1
***Oxido-reductases***	**58**	**23**	**19**
Class III peroxidase subfamily	26	9	6
Laccases	5	1	1
BBE (S)-reticulins	6	3	3
Multicopper oxidases	12	6	5
Copper amine oxidases	2	0	0
Thiol reductase (GILT family)	1	0	0
Expressed protein (glyoxal oxidase domain/DUF1929)	1	0	0
Expressed protein (thioredoxin fold)	1	1	1
Expressed proteins (GMC oxido-reductase domain)	2	1	1
Expressed protein (DUF568)	0	2	2
Cytochrome b5-like Heme/Steroid binding domain	2	0	0
***Proteins related to lipid metabolism***	**39**	**12**	**8**
lipid-transfer proteins (LTPs)	10	1	1
GDSLs	14	4	2
GDPDs	3	2	2
MD-2-related lipid-recognition (ML) domain	1	0	0
Phosphoesterases	2	0	0
Expressed protein (lipase/lipooxygenase domain, PLAT/LH2)	4	0	0
Phospholipase C	1	2	1
Phosphodiesterase/phosphate transferase	1	1	1
Lecithin	1	0	0
Ceramidase	1	1	1
BPI/LBPs	1	1	0
***Miscellaneous proteins***	**61**	**23**	**9**
Thaumatins (PR5)	5	3	1
Germins	5	0	0
Metallophosphoesterases (PAPs)	10	12	5
Blue copper binding proteins	8	1	0
Dirigent proteins	6	3	1
Phosphate-induced (phi) proteins	3	0	0
SCP-like extracellular proteins (PR-1)	2	0	0
Phosphorylases	3	1	0
Strictosidine synthases	1	0	0
Gibberellic acid-stimulated Arabidopsis (AtGASA1) proteins	3	0	0
Homologous to dienelactone hydrolase	1	1	0
Aldose-1-epimerases	2	0	0
Homologous to phosphatidylinositol transfer protein	1	0	0
Hexokinase	1	0	0
Glucose/sorbosone dehydrogenaes	1	0	0
Carbonic anhydrases	3	0	0
Expressed proteins (cupin domain)	5	2	2
Expressed proteins	1	0	0
***Unknown function***	**50**	**21**	**9**
Expressed proteins (Gnk2-homologous domain, antifungal protein of Ginkgo seeds)	3	4	2
Expressed proteins (DPBB domain)	4	0	0
Expressed proteins (DUF642)	2	0	0
Plant basic secretory protein (BSP) family proteins	2	0	0
Expressed protein (alpha/beta hydrolase fold)	1	0	0
Expressed proteins (WD40-like beta propeller domain)	3	1	1
NADPH-dependent FMN reductases	2	0	0
Homolog TC173720	2	0	0
Expressed proteins (PA domain)	3	1	1
Expressed proteins (glyoxal oxidase domain/DUF1929)	2	0	0
Expressed proteins (saposin domains)	2	1	1
Expressed proteins (Ole e1 allergen domain)	2	0	0
Expressed protein (cyclase domain)	1	2	1
Expressed protein (BURP domain)	1	0	0
Expressed protein (Xylose isomerase-like TIM barrel)	1	1	1
Expressed protein (human brain CREG protein domain)	1	0	0
Expressed protein (ferritin-like domain)	1	0	0
Expressed protein (DUF303)	1	0	0
Expressed protein (DUF538)	1	0	0
Expressed protein	15	11	2
***Structural proteins***	**4**	**2**	**1**
LRR-extensins	3	1	1
homologous to AGP/proline-rich protein	1	0	0
hydroxyproline-rich glycoprotein	0	1	0

### Functional classification of CWPs

To better understand the biological functions of CWPs, CWPs were categorized on the basis of their functional domains proposed by Jamet et al. [6]. A total of 578 CWPs (501 + 195–118) were divided into nine groups (Fig. 2B). Among them, proteins acting on polysaccharides (PACs; 147) were the largest functional class, occupying 25.4% of total CWPs. Proteases (Ps; 94) were the second largest class, accounting for 16.3% of total CWPs. Oxido-reductases (ORs; 62) were the third largest class, occupying 10.7% of identified CWPs, followed by proteins involved in signaling (PSs; 56), proteins related to lipid metabolism (PLMs; 43), and proteins with interaction domains (PIDs;34), accounting for 9.7, 7.4 and 5.9% of the identified CWPs, respectively. Structural proteins (SPs; 5, 0.9%) had lowest abundance, only containing 5 members. The remaining CWPs related to various functions were categorized as miscellaneous proteins (MPs; 75, 13.0%), and CWPs with previously uncharacterized domains were referred to as proteins of unknown function (PUFs; 62, 10.7%).

### CWP comparison between *C. sinensis* and other two species

As expected, the functional distribution of CWPs identified from *C. sinensis* leaves was in good concordance with that from *A. thaliana* rosettes and *B. distachyon* leaves (Additional file 5: Fig.S1) with PACs, Ps, and ORs representing top three functional classes. Notably, the percentage of PSs in *C. sinensis* (9.7%) leaves was obviously higher than that in *A. thaliana* rosettes (3.7%) and *B. distachyon* (4.0%) leaves, respectively [12, 20]. Such a difference may be attributed to the longer lifecycle of the woody evergreen leaf in *C. sinensis.*

### Main representative of functional classes

**Fig. 3:**
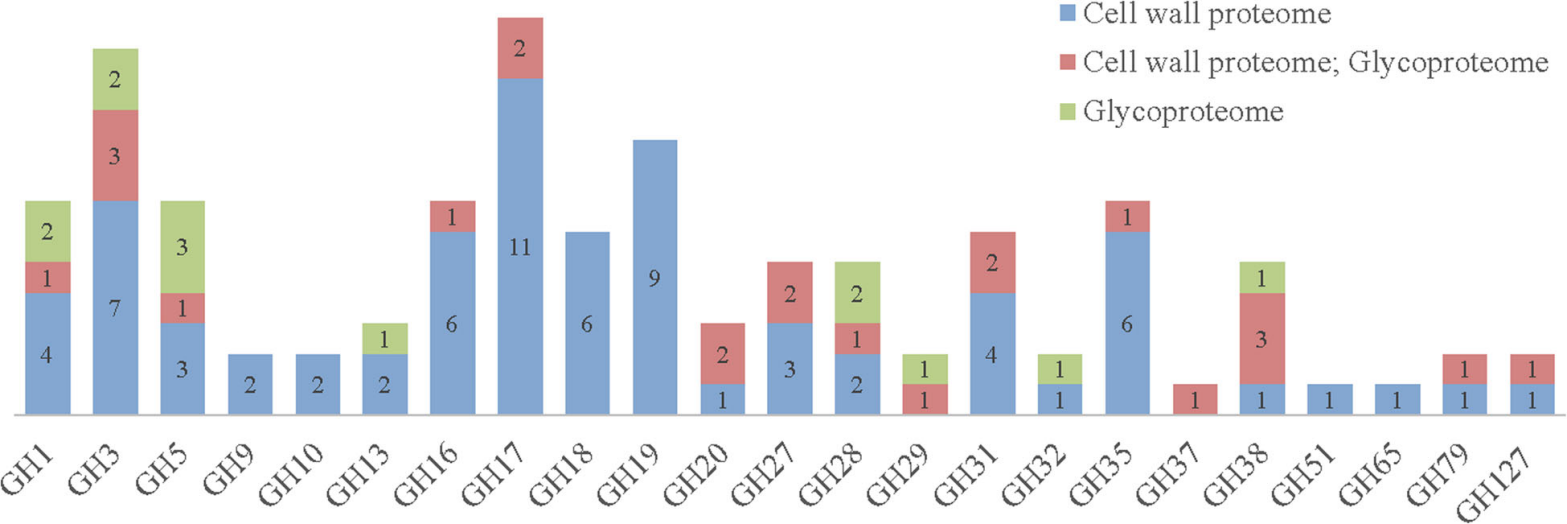
Glycoside hydrolases identified from *C. sinensis* leaves

In PAC class, GHs are the major representative (Table 1). In this study, a total of 110 GHs were identified accounting for 74.8% in PAC class, which fell into 23 families including GH1, GH3, GH5, GH9, GH10, GH13, GH16, GH17, GH18, GH19, GH20, GH27, GH28, GH29, GH31, GH32, GH35, GH37, GH38, GH51, GH65, GH79, and GH127 according to CAZy nomenclature based on sequence homology (Fig. 3). As expected, the most representative families were GH3 and GH17, as previously documented [12, 20, 26]. Moreover, GH1, GH5, GH16, GH18, GH19, GH27, GH28, GH31, GH35, and GH38 were also well representative families with at least 5 members in each family (Fig. 3). In addition, less representative CWPs acting on polysaccharides were also identified, including carbohydrate esterase [11 pectinesterases (known as pectin methylesterases (PMEs)) and 3 pectinesterase inhibitors (PMEIs)], 4 glycosyl transferases (GTs, including GT2, GT31, GT48, and GT68), 6 expansins, 4 PNGase A, 3 pectin acetylesterases (PAEs), 2 pectate lyases (PLs), and 4 carbohydrate acylation (trichome birefringence-like proteins).

In Ps class, Asp proteases (28), Ser carboxypeptidases (28), Ser proteases (19), and Cys proteases (14) are main families, occupying 94.7% of Ps. ORs functional class mainly comprised of class III peroxidase (PODs, 29), multicopper oxidases (13), BBE (berberine bridge enzyme) (S)-reticulin (6), and laccases (5). Other CWPs related to redox processes were identified including monocopper oxidase-like proteins (SKU5 and SKS1), blue copper proteins, and ascorbate oxidases.

PSs class mainly contained fasciclin-like arabinogalactan proteins (FLAs, 9) and receptor-like protein kinases (RLKs) superfamily proteins (38). Among them, RLKs comprised 21 LRR-RLKs, 6 cysteine-rich recetor-like protein kinases, 3 S-locus receptor kinase subfamily proteins, 2 wall-associated receptor kinases, and 6 lectin receptor kinase subfamily proteins. PLMs class mainly consisted of lipid-transfer proteins (LTPs, 10) and GDSL esterase/lipases (GDSLs, 16). As for SPs class, only five CWPs were identified in this study including 3 leucine-rich repeat extensin-like proteins (LRR-EXTs), 1 non-classical arabinogalactan protein 31-like (AGP), and 1 hydroxyproline-rich glycoprotein (HPRG). Identified MPs mainly encompasses purple acid phosphatases (PAPs, 17), blue copper binding proteins (BCPs, 9), dirigent proteins (DIRs, 8), germin-like proteins (GLPs, 5), thaumatins (7), and proteins with a cupin domain (5).

## Discussion

### Identification and functional classification of identified CWPs

Totally, 3618 TCWPs were identified in *C. sinensis* leaves by sequential salt extractions and UPLC-MS/MS. Among them, 627 TCWPs were homologs of the CWPs in WallProtDB database, whereas 501 TCWPs were in good accordance with the CWPs defined by multiple bioinformatics analyses. In except for firstly defined CWPs, there is an identification difference between WallProtDB database and bioinformatics analyses, which might ascribe to a low homology of CWPs between *C. sinensis* and other plant species indexed in WallProtDB.

Finally, 501 TCWPs and 3079 TCWPs were designated as CWPs and intracellular proteins, indicating that TCWPs were subjected to the contamination during TCWPs preparation. Similar high contamination of intracellular proteins was also detected in sugarcane [25] and rice [29], accounting for 81.6 and 80.5%, respectively. So far there have been rare cell wall proteomics studies, therefore CWP extraction remains to be improved. In spite of the high contamination of intracellular proteins, this study adopted cell wall proteomics to enhance the CWP coverage in *C. sinensis*.

At the same time, most identified N-glycoproteins (195, 74.4%) were targeted into the cell wall/extracellular/plasma membrane in *C. sinensis*, and thus they were designated as CWPs. Our results agreed well with those of the studies of tomato fruit [35] and *B. distachyon* leaf [43], in which 65 and 60% of N-glycoproteins were found to be located in the apoplast/cell wall/plasma membrane, respectively, demonstrating that N-glycoproteomics is a feasible method to identify and characterize CWPs.

It should be noted that 25 CWPs were newly identified ones in this study (Additional file 3: Table S3), and that more CWPs were identified through cell wall proteomics (501 CWPs) than N-glycoproteomics (195CWPs), indicating cell wall proteomics was more effective than N-glycoproteomics for CWP identification. However, the use of N-glycoproteomics as supplementation can further enhance CWP identification effect. Considering this, we propose that combined cell wall proteomics and N-glycoproteomics during CWP identification and characterization.

### Possible functions of identified CWPs

#### Identified CWPs acting on cell wall polysaccharides

##### Glycoside hydrolases (GHs)

GHs are the overwhelming majority of identified CWPs with 19.0%. Possible substrates of most GH families are hemicelluloses (xyloglucan, xylans, glucomannans) and pectin (galactans, homogalacturonan). Of GHs identified in this study, GH16, GH29, GH31, and GH65 potentially act on xyloglucans; GH10 and GH51 show a possible action on xylans; and GH28 and GH35 can hydrolyze homogalacturonan and galactans, respectively [23, 44, 45] (Additional file 6: Table S5). Moreover, GH1, GH3, and GH5 possess broad substrates range, and their enzymes are reported to be involved in the modification and/or breakdown of cell wall hemicelluloses and pectins [46, 47], and to participate in lignification and secondary metabolism [48]. Identification of these GH families suggested that hemicelluloses and pectins might undergo important structural changes in the leaves of *C. sinensis*. Furthermore, GH127 (also known as DUF1680 domain protein), recently characterized as a novel β-L-arabinofuranosidase, might be involved in the degradation of cell wall polysaccharides and hydroxyproline-rich glycoproteins [49], and GH9 was known to catalyze the endohydrolysis of cellulose.

Some identified GHs might participate in defense against pathogens and various stresses. Chitin and β-1,3- or β-1,6-glucan are main components of cell walls of various fungi. GH17 acts as β-1,3-glucanase; GH18 and GH19 act as chitinases; and GH20 functions as key hydrolyzation enzyme of chitin, and these four GHs possess antifungal activity to degrade fungus cell walls and participate in defense against pathogens [45, 50]. Chitinase has been reported to respond to abiotic stress [42, 51]. GH37, a non-reducing sugar, was identified as a new CWP in this study and it has been found to be a universal stabiliser of protein conformation and probably contribute to various stress defense [52].

Several identified GHs including GH13, GH27, and GH32 might be implicated in the mobilization, allocation, and partitioning of storage reserves. GH13 is involved in the hydrolysis of starch and glycogen to yield glucose and maltose [53]. GH27 is one of three hydrolyzing enzymes of galactomannans (cell wall storage polysaccharide) [54], and GH32, as an invertase, is involved in long-distance nutrient allocation and carbohydrate partitioning [55, 56]. Additionally, a couple of GH enzymes including GH3, GH18, GH19, GH35, GH38, and GH79 are involved in post-translational modifications (PTMs) of glycoproteins [32, 45]. In this study, GH3, GH35, GH38, and GH79 were verified as N-glycoproteins.

Collectively, a large number of GHs associated with cell wall metabolism and defense were identified in this work, which is consistent with previous reports of sugarcane stems and leaves [26], *B. distachyon* grains [21], *S. officinarum* cell suspension [25]. Our data reveal the potential functions of identified GHs such as complex cell wall carbohydrate remodeling, pathogen and stress response, mobilization and allocation of storage reserves, and glycoprotein PTMs. Our results might be attributed to sustainable remodeling during plant growth and development and terrestrial habit of plants.

##### Other CWPs acting on polysaccharides

PMEs, PAEs, and PLs are pectin-modifying enzymes. PMEs catalyse the demethyl-esterification of homogalacturonan domain of pectin [57]. The degree of pectin methylation/demethylation affects cell wall stiffening and access to enzymes [58]. Demethyl-esterificated pectin favors the cleavage of the acidic polygalacturonic chains by GH28 and PLs. Likewise, PAEs can regulate pectin deacetyltation by cleaving the acetylester bond from pectin [59]. Overall, these enzymes play a major role in controlling cell wall plasticity/rheology by affecting pectin metabolism [60].

Trichome birefringence-like proteins and PNGase A are also two modification enzyme families in cell wall. The former is characterized as xylan acetyltransferases, and it is associated with the xylan O-acetylation mediation, secondary wall deposition, and pathogen resistance [61]. The latter is one of deglycosylation enzyme, and it is involved in the release of N-glycans from glycopeptides generated by the proteolysis of denatured glycoproteins [62].

Expansin, known as non-enzymatic protein and the most important structural protein, plays a central role in cell wall extension via their action on the cellulose-hemicellulose network, suggesting that expansin is essential for primary cell wall structure during plant growth- and development-related processes [63]. In addition, 4 cell wall GT families might be associated with the biosynthesis of cell wall polymers.

#### Identified CWPs functioning as proteases

Proteases are necessary for protein turnover, maturation of enzymes, and defense against pathogens [45, 64]. Consequently, plant proteases localized in the cell wall might be responsible for CWP degradation or maturation, and further they might play crucial roles in a striking variety of biological processes such as plant growth and branching, flower time regulation, and in defense responses.

#### Identified CWPs involved in redox

Class III PODs, a large multigene families, accounted for one half of OR functional class. Class III PODs are involved in lignin metabolism by catalyzing the oxidative polymerization of monolignols [65], stress responses, and signaling transduction via consuming hydrogen peroxide and generating reactive oxygen species [66]. Class III PODs can mediate cross-linking of cell wall compounds such as structural proteins, monolignols, and aromatic amino acids containing polysaccharides [67–69]. Like class III PODs, laccases are candidates for polymerizing monolignol unit into lignin, suggesting that laccases are essential for cell wall lignification [70, 71]. BBE-like proteins, as monolignol oxidoreductases, may participate in the mobilization and oxidation of monolignols required for polymerization processes [72]. Overall, three highly representative enzyme families in the redox class were considered to be involved in ligin production and subsequent the reinforcement of cell wall strength and rigidity, which supported plant defense against adverse environmental factors.

In addition, monocopper oxidase-like proteins (SKU5 and SKS1), blue copper proteins, and ascorbate oxidases were found, they might play a role in both cell wall loosening, expansion, and reticulation processes [24, 73].

#### Identified CWPs involved in signaling transduction

In this study, identified signaling transduction-related CWPs mainly consisted of FLAs and RLKs superfamily proteins. FLAs, heavily O-glycosylated CWPs, have been found to be correlated with cell wall formation [74], cell-to-cell adhesion and communication [75], and abiotic stress response [76]. RLKs, as primary cell wall “sensors”, are responsible for controlling diverse signaling events [77]. RLKs possess important functions in a wide variety of development- and defense-related processes, for example, they can recognize extracellular ligand to activate the intracellular kinase domain, resulting in downstream signaling transduction [78].

#### Identified CWPs related to lipid metabolism

CWPs in the functional class have been reported to be related to lipid metabolism [79–83]. LTPs are required for lipid export to the cell surface, and they are closely associated with cutin and wax formation [79]. One LTP has been found to be involved in cell wall extension by interacting with the cellulose/xyloglucan network [80]. GDSLs, a recently discovered subclass of lipolytic enzymes, possess multifunctional properties, and play important roles not only in the formation of surface cutin and epi-cuticular wax [81], but also in the tolerance to biotic and abiotic stresses [82, 83]. In summary, numerous LTPs and GDSLs might play important roles in cuticle assembly during the growth and development of *C. sinensis* leaf. The identification of CWPs related to PLMs is conducive to our understanding of leathery leaf of *C. sinensis*.

#### Structure proteins

Due to be still resistant to salt-extraction, structure proteins were eluted difficultly so far. In this study, five structure proteins were identified. LRR-EXTs have been reported to influence mechanical properties of cell wall by forming insolubilized, covalently crosslink with cell wall components [84], and they can perceive extracellular signals and indirectly relay them into the cytoplasm to regulate plant growth and salt tolerance, and consequent they are important for cell wall development, plant growth, and stress tolerance [85]. Non-classical AGPs have both proline-rich domain and non-proline-rich domain, may function in metal ion-binding, defense response, and they can interact with pectin [86, 87]. HPRG is an important structural components of plant cell walls, and are related to structural integrity, cell-cell interaction, and intercellular communication [88].

#### Identified CWPs related to other functions

Regarding several MPs-related CWPs, PAPs might be associated with the degradation of xyloglucan and oligosaccharides via dephosphorylating CWPs such as alpha xylosidase and β glucosidase [89]. DIRs are related to lignin polymerization [90, 91], and they play important roles in various stress responses and cell wall modification/reinforcement during cell wall integrity maintenance [92]. BCPs, GLPs, cupins, and Thaumatins have been previously reported to be associated with stress responses in plants [93–96].

Several enzymes of CWP inhibitor were also detected in this study, including PMEIs, PGIPs (polygalacturonase inhibitor-like), and Cys proteinase inhibitor. PMEIs partially inhibit the activity of PMEs and adjust the degree of pectin methyl-esterification. PGIPs specifically bind with polygalacturonases (GH28), thereby inhibiting the hydrolyzation of pectin and regulating pectin degradation, eventually triggering defense response against microbes and insects [97]. In summary, two couples (PMEIs and PME, PGIPs and PG) occurred coincidentally, and they modulate precisely pectin metabolism. Cys proteinase inhibitor exhibit inhibitory activities against specific Cys proteases, thus might function in preventing insect predation [98].

### Roles of various CWPs in plant cell wall formation and defense response

**Fig. 4:**
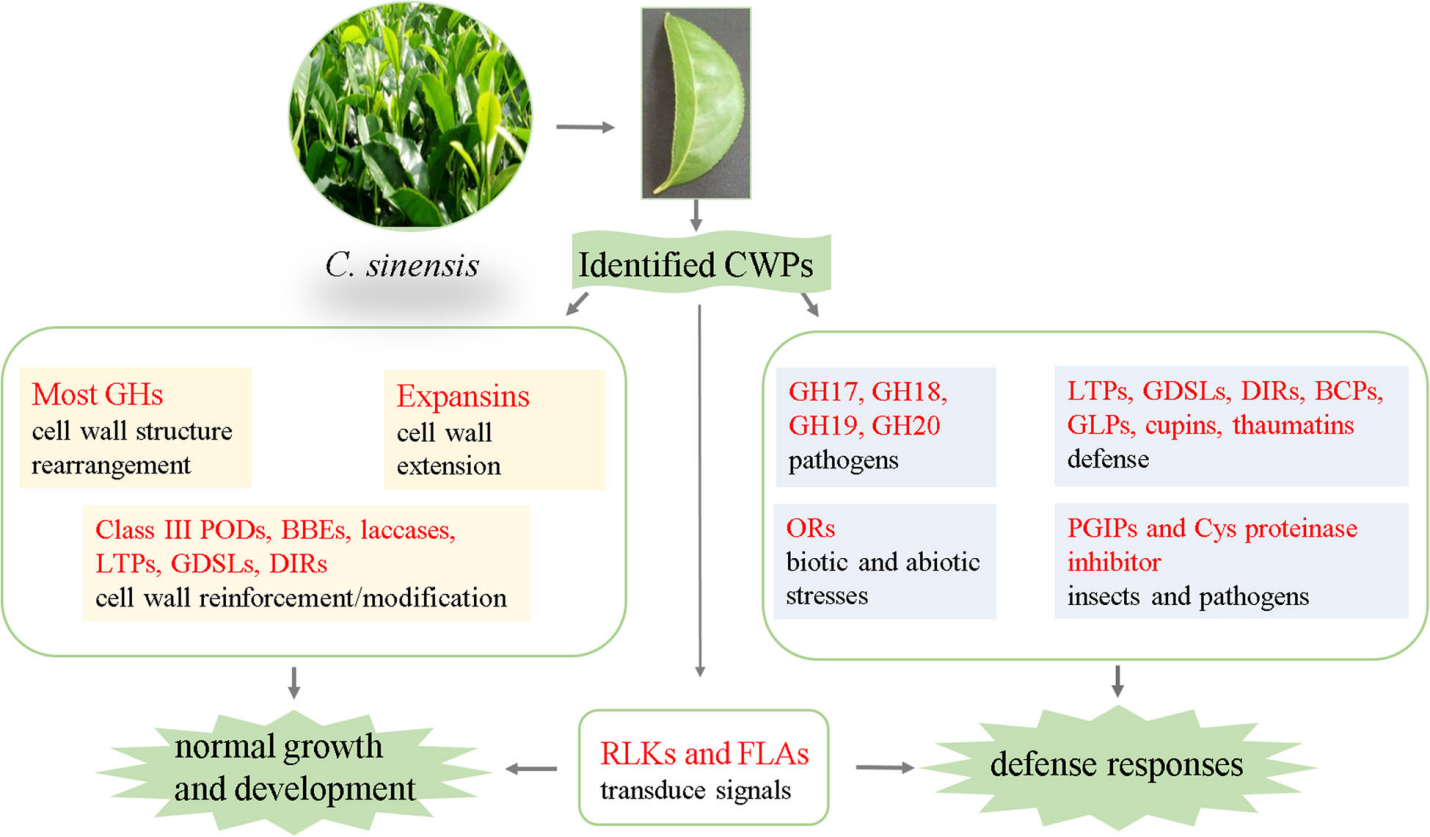
A work model of CWPs identified from *C. sinensis* leaves. The figure is under copyright, each researcher can use and adapt it by citing our paper

Under dynamic environmental conditions, plants grow and develop continuously, and they always encounter various stresses and deleterious attack from insects and microbes. Plant cell walls, as the first barrier, change constantly to be adapted to environmental stresses. Doubtlessly, CWPs play central roles in altering cell wall properties. On basis of our results, a work model of identified CWPs mainly related to plant cell wall formation and defense response was proposed (Fig. 4).

To satisfy the requirement of normal growth and development, a large number of CWPs are activated to adjust cell wall structure. In this study, numerous CWPs related to PACs were identified, mainly including GH1, GH3, GH5, GH9, GH10, GH16, GH28, GH29, GH31, GH35, GH51, and GH65, and they might contribute to the rearrangement of cell wall structure, while expansin probably give rise to cell wall extension. Several CWPs associated with the formation and metabolism of secondary cell wall, such as class III PODs, BBEs, laccases, LTPs, GDSLs, and DIRs, may favor the reinforcement/modification of cell wall (Fig. 4).

Confronted with adverse environment, *C. sinensis*, a terrestrial plant, has no ability to escape. Therefore, it has evolved some mechanisms of defense responses such as altering cell wall properties. Many CWPs identified in this study are potentially involved in various defense. GH17, GH18, GH19, and GH20 have been reported to be involved mainly in response to pathogen stress as well as abiotic stress by hydrolyzing chitin. Class III PODs, monocopper oxidase-like proteins, blue copper proteins, and ascorbate oxidases are involved in response to various biotic and abiotic stresses by redox reaction. LTPs, GDSLs, and DIRs are also associated with defense response through the regulation of secondary cell wall. PGIPs and Cys proteinase inhibitor might function in improving protection against insects and pathogens [99] via inhibiting the activity of degradation enzymes of invaders. Likewise, BCPs, GLPs, cupins, and thaumatins also function in defense response (Fig. 4).

To sense dynamic environment and changing complex cell wall structures, plants have developed cell wall integrity-sensing pathway to transduce signals into cytoplasm. A number of sensors on the plasma membrane including RLKs and FLAs were identified in present study, which can mediate cross-talk between the cell wall and the cytoplasm in *C. sinensis* (Fig. 4).

## Conclusions

This study combined cell wall proteomics and N-glycoproteomics to identify CWPs in *C. sinensis*. A total of 3880 proteins were identified by sequential salt extraction and UPLC-MS/MS. Meanwhile, 262 N-glycoproteins were identified by HILIC enrichment coupled with UPLC-MS/MS. Subsequently, 501 out of 3880 proteins and 195 out of 262 N-glycoproteins were designated as CWPs by multiple bioinformatics analysis. Of these designated CWPs, 118 were in common. In total, 578 CWPs were identified from *C. sinensis* leaves, 25 of which were determined as newly identified CWPs. This study was the first attempt of large-scale investigation of CWPs by cell wall proteomics and N-glycoproteomics in *C. sinensis.* It provides a reference for using a combined strategy of cell wall proteomics and N-glycoproteomics to improve CWP identification and characterization. Our findings promote the understanding of cell wall formation and defense response in *C. sinensis*.

## Methods

### Plant materials

From the tree top, the first to fifth leaves were collected from 20 uniform 2-year-old cutting seedlings of the Echa 1 variety (*C. sinensis* cv. ‘Echa 1’) in tea germplasm bank located in Wuhan city of Hubei province (China). The collected leaves were washed three times with Milli-Q water, and ground into fine power in liquid nitrogen immediately, and finally stored at − 80 °C for subsequent use.

### Cell wall enrichment

Cell wall fractions were obtained from the leaves of *C. sinensis* using sequential washes as described by Printz et al. [100] with slight modification. Briefly, 5 g fine power of the leaves were homogenized with 3-fold volumes of 0.4 M sucrose buffer for 10 min, vortexed for 2 min, shaken overnight at 250 rpm at 4 °C, and then centrifuged. Subsequently, 0.6 M sucrose buffer was added into the precipitations, shaken for 30 min at 250 rpm at 4 °C and centrifuged. Afterwards, 1 M sucrose buffer was added into the precipitations again, suspended, and centrifuged. Finally, the precipitations were washed twice using 5 mM sodium acetate buffer. The final precipitations were cell wall fractions (pellet). Sucrose buffer contained 5 mM sodium acetate and 1% protease inhibitor cocktail (ApexBio). All the buffers (pH 4.6) were precooled at 4 °C, and the centrifugation was performed at 1000 rpm for 15 min at 4 °C.

### Cell wall protein extraction

CWPs were extracted successively using CaCl_2_, EGTA, and LiCl according to the method reported by Printz et al. [100]. Briefly, 0.2 M CaCl_2_ buffer was firstly added into cell wall pellet, shaken for 30 min at 200 rpm at 4 °C, followed by centrifugation. Then the supernatants were collected. This process was repeated once, and the supernatants were pooled as CaCl_2_ fractions. Afterwards, cell wall pellets were mixed with 50 mM EGTA buffer, followed by shaking for 1 h at 300 rpm at 37 °C, centrifugation and supernatant collection. This procedure was repeated twice, and all the supernatants were collected as EGTA fractions. Cell wall pellets were finally re-suspended in 3 M LiCl buffer, homogenized overnight at 250 rpm, 4 °C. After centrifugation, the supernatants were collected once again. The CWPs were once again extracted from the cell wall pellets with 3 M LiCl buffer by shaking for 6 h at 250 rpm, 4 °C. The obtained supernatants were pooled and stored as LiCl fractions. Finally, CaCl_2_, EGTA, and LiCl fractions were combined as target CWPs (TCWPs) fractions. All extraction buffers were precooled at 4 °C, and the centrifugation was performed for 15 min at 10000 rpm at 4 °C.

### Whole protein extraction

Whole proteins (WPs) were extracted from *C. sinensis* leaves according to several previous reports [42, 101, 102]. Briefly, about 0.5 g fine powder was firstly homogenized with 5 ml pre-cooled homogenization buffer [containing 20 mM Tris-HCl (pH 7.5), 250 mM sucrose, 10 mM EGTA, 1 mM PMSF, 1 mM DTT, and 1% (v/v) Triton], and then centrifuged at 12000 g for 20 min at 4 °C. The obtained supernatants were pooled and stored as whole protein fractions.

### Protein precipitation and cleaning

According to our previous study [42], WP fractions and TCWP fractions were precipitated severally by Tris-phenol (pH ≥ 8.0) and ammonium acetate. In brief, the fractions were mixed with equal volume of Tris-phenol, and vortexed, followed by centrifugation at 12000 g for 20 min at 4 °C. Afterwards, the phenol phase was transferred carefully into other tubes, mixed thoroughly with 5 volumes of 0.1 M ammonium acetate in 100% methanol, and incubated at − 80 °C overnight. The precipitated proteins were washed twice with 0.1 M ammonium acetate and acetone, separately. The protein pellets were lyophilized and then dissolved into lysis buffers [containing 7 M urea, 2 M thiourea, 4% CHAPS, 250 mM DTT, and 0.2% (v/v) Bio-Lyte]. Protein concentration was determined with BCA kit according to the manufacturer’s instructions.

### Protein digestion

Before trypsin digestion, WPs and TCWPs were reduced with 5 mM dithiothreitol for 30 min at 56 °C, and alkylated with 11 mM iodoacetamide for 15 min at room temperature in darkness, followed by urea dilution to concentration < 2 M through the addition of 100 mM triethylammonium bicarbonate. Afterwards, WPs and TCWPs were digested firstly by trypsin (1:50 trypsin/protein) overnight at 37 °C, and then by trypsin (1:100 trypsin/protein) for 4 h. Finally, tryptic peptides were desalted by Strata X C18 SPE column (Phenomenex, USA) and concentrated by centrifugal concentrator.

### HPLC fractionation

After tryptic digestion, the peptides from WPs and TCWPs were fractionated severally by the use of high pH reversed-phase HPLC (high-performance liquid chromatography) with Agilent 300 Extend C18 column (5 μm particles, 4.6 mm inner diameter, and 250 mm length). Briefly, the digested peptides were first separated into 60 fractions with a gradient of 8 to 32% acetonitrile (pH 9.0) for more than 60 min. Subsequently, the peptides were pooled into 4 fractions and dried by vacuum centrifugation for further use.

### Affinity enrichment of N-glycopeptides

To enrich N-glycosylation peptides, the dried peptides from WPs were firstly dissolved in 40 μL enrichment buffer (containing 80% acetonitrile and 1% trifluoroacetic acid), and then loaded into HILIC micro-column to separate glycopeptides from non-glycopeptides by centrifugation for 15 min at 4000 g. To remove non-specifically adsorbed peptides, HILIC micro-column was washed three times with enrichment buffers. Subsequently, the bound peptides were eluted from the micro-column with 10% acetonitrile, and then vacuum-dried. The lyophilized N-glycopeptides were reconstituted in 50 μL NH_4_CO_3_ buffer (50 mM) in heavy oxygen water and incubated with 2 μL PNGase F at 37 °C overnight. Finally, the resultant N-glycopeptides were desalted with C18 ZipTips (Millipore) according to the manufacturer’s instructions and lyophilized for LC-MS/MS analysis.

### UPLC-MS/MS analysis

For LC-MS/MS analysis, the peptides were firstly dissolved in solvent A (containing 0.1% (v/v) formic acid and 2% acetonitrile), and then gradient-eluted in EASY-nLC 1000 UPLC system. Peptide separation was conducted with home-made reversed phase column (25 cm length, 100 μm ID). TCWP peptides were gradient eluted as follows: 450 nL/min constant flow; starting with 7% ~ 25% solvent B (containing 0.1% formic acid in 90% acetonitrile) for 0–40 min, followed by 25% ~ 35% solvent B for 40–52 min, 35% ~ 80% solvent B for 52–56 min, and 80% solvent B for 56–60 min. Deglycosylated peptides were gradient eluted with following procedures: 500 nL/min constant flow, starting with 4 to 20% solvent B for 0–24 min, 20 to 32% solvent B for 24–32 min, 32 to 80% solvent B for 32–36 min, and finally maintaining in 80% solvent B for 36–40 min.

Subsequently, the separated TCWP peptides and deglycosylated peptides were respectively injected into a nanoelectrospray ion source, followed by MS/MS analysis in Q Exactive™ and Orbitrap Fusion mass spectrometer (Thermo Fisher scientific). Briefly, the applied electrospray voltage was 2.0 kV, and the intact peptides and their secondary fragments were detected and analyzed by Orbitrap with a data-dependent acquisition mode automatically switching between MS scan and MS/MS scan.

TCWP peptides were fully scanned at a resolution of 70,000 with m/s scan range of 350–1800. Afterwards, the top 10 most intense parent ions per scan were selected for higher-energy collisional dissociation fragmentation (HCD) at 28% collision energy. The generated fragments were further analyzed at a resolution of 17,500 with a fixed first mass of 100 m/z. To increase the effective utilization rate of mass spectrometry, the related parameters were set as follows: automatic gain control of 5E4, 30 s dynamic exclusion, 100 ms maximum inject, and signal threshold of 20,000 ions/s. Likewise, deglycosylated peptides were fully scanned at a resolution of 60,000 with m/s scan range of 350–1550. The top 20 most intense parent ions per scan were selected for HCD at 35% collision energy, and then the resultant fragments were analyzed at a resolution of 15,000 with a fixed first mass of 100 m/z. Similarly, the related MS parameters were set as follows: automatic gain control of 5E4, 15 s dynamic exclusion, 200 ms maximum inject and signal threshold of 5000 ions/s were used.

### Database search

The resultant raw MS/MS data were processed using MaxQuant search engine (v.1.5.2.8) with the following query parameters: (i) tea tree genome database (Camellia_sinensis_4442 with 53,512 sequences [103];) concatenated with reverse decoy database and mass spectrometry contaminants database for MS/MS search; (ii) Trypsin/P for enzyme cleavage and 2 missing cleavages; (iii) mass tolerance of 20 ppm and 5 ppm for peptide ions in first search and main research, respectively, and 0.02 Da for fragment ions; (iv) length of 7 amino acid residues as minimum peptide length, and 5 as maximum modification number in a peptide; (v) Cysteine alkylation as fixed modification; (vi) Variable modification: methionine oxidation and N-terminal acetylation for TCWPs, and methionine oxidation and deamidation (NQ), asparagine deamidation (^18^O)for N-glycoproteins; (vii) FDR ≤ 1% for protein identification and peptide-spectrum match identification.

### Multiple bioinformatics analyses

CWPs were predicted and functionally categorized using WallProtDB database [104]. Glycoside hydrolases and carbohydrate esterase were grouped according to CAZy database [105]. N-terminal signal peptide of identified proteins was predicted using SignalP [106]. Transmembrane domain was evaluated by TMHMM server [107]. Subcellular localization predication was performed using TargetP [108], WoLF PSORT [109], Loctree 3 [110], and Plant-mPLoc [111]. ER retention signal was checked using Prosite [112].

## Supplementary Information

**Additional file 1: Table S1.** Proteins identified from *C. sinensis* leaves by cell wall proteomics.

**Additional file 2: Table S2.** N-glycoproteins identified from *C. sinensis* leaves.

**Additional file 3: Table S3.** 578 CWPs identified from *C. sinensis* leaves by cell wall proteomics and N-glycoproteomics.

**Additional file 4: Table S4.** Plasma membrane proteins identified by cell wall proteomics.

**Additional file 5: Fig.S1.** Functional categories of CWPs identified from *C. sinensis* leaves, *B. distachyon* leaves, and *A. thaliana* rosettes.

**Additional file 6: Table S5.** Possible substrates of GHs identified from *C. sinensis* leaves.

## Data Availability

All generated or analyzed data were included in this published article, and all relevant raw data were deposited in ProteomeXchange Consortium with the dataset identifier PXD026772.

## Abbreviations

CWPs: Cell wall proteins
TCWPs: Target cell wall proteins
WPs: Whole proteins
GHs: Glycoside hydrolases
PACs: Proteins acting on polysaccharides
Ps: Proteases
ORs: Oxido-reductases.
PSs: Proteins involved in signaling.
PLMs: Proteins related to lipid metabolism.
PIDs: Proteins with interaction domains.
PODs: Peroxidases.
