# Supplementary figures and images for: Investigation of cell wall proteins of C. sinensis leaves by combining cell wall proteomics and N-glycoproteomics
Source: BMC Plant Biol. 2021 Aug 20;21:384. doi: 10.1186/s12870-021-03166-4 (PMC8377857; doi:10.1186/s12870-021-03166-4)

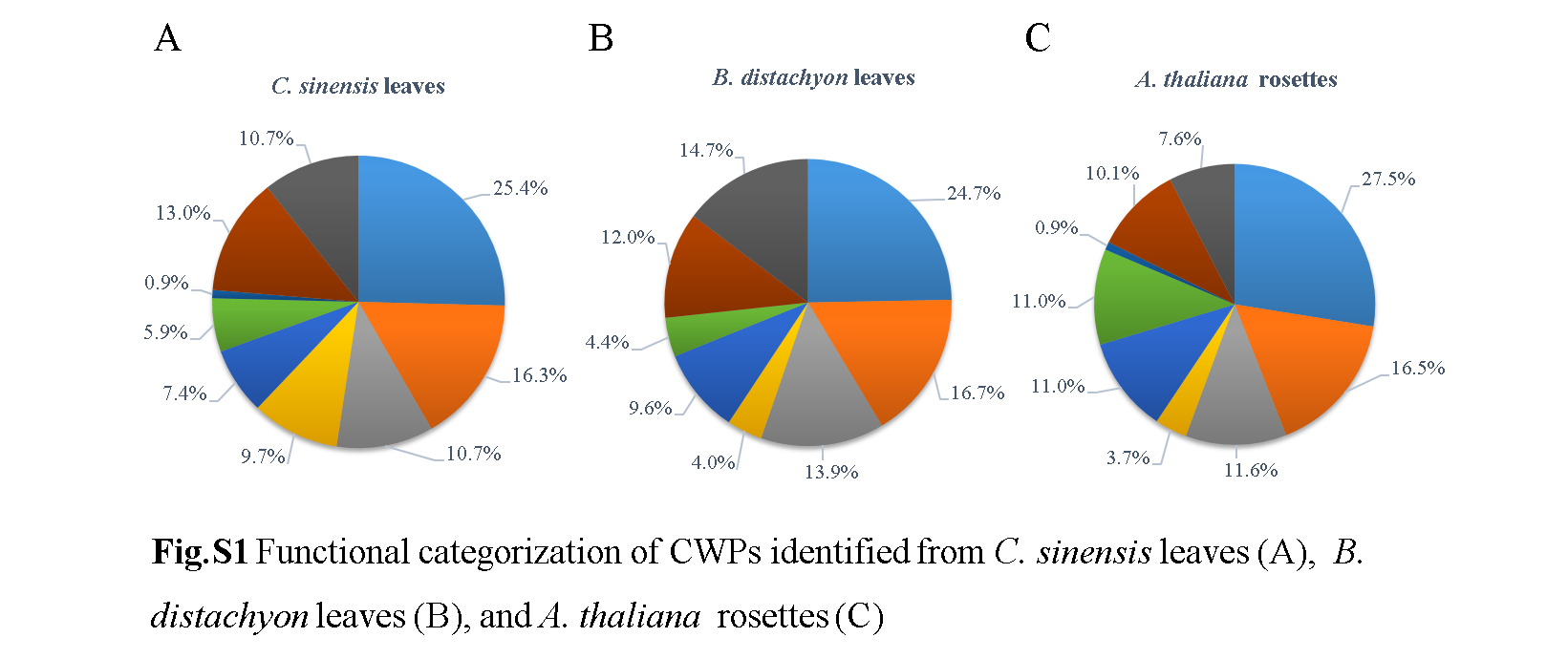

Supplement: Supplementary file 5 — Additional file 5: Fig.S1. Functional categories of CWPs identified from C. sinensis leaves, B. distachyon leaves, and A. thaliana rosettes. [file 12870_2021_3166_MOESM5_ESM.tif]
