# Supplementary material for: Investigation of cell wall proteins of C. sinensis leaves by combining cell wall proteomics and N-glycoproteomics
Source: BMC Plant Biol. 2021 Aug 20;21:384. doi: 10.1186/s12870-021-03166-4 (PMC8377857; doi:10.1186/s12870-021-03166-4)
Supplement: Supplementary file 6 — Additional file 6: Table S5. Possible substrates of GHs identified from C. sinensis leaves. [file 12870_2021_3166_MOESM6_ESM.docx]

**Table 5** Possible substrates of GHs identified from *C. sinensis* leaves

| GH family | Identified proteins | Enzyme activity | Possible substrates |
| --- | --- | --- | --- |
| GH1 | β-glucosidase | β-glucosidase; cellobiose; β-mannosidase; glucosidase | glucan, xyloglucan, cellulose |
| GH3 | β-D-xylosidase; β -xylosidase /α-L-abinofuranosidase | β-D-xylosidase; α-L-arabinofuranosidase | xylan, arabinoxylan, arabinan |
|  | β-glucosidase BoGH3B | β-glucosidase | glucan, xyloglucan, cellulose |
| GH5 | glucan 1,3-β-glucosidase; | β-glucanases | β-glucan |
|  | mannan endo-1,4-β-mannosidase | β-mannanase | mannans, galactomannans, glucomannans |
| GH9 | endoglucanase | cellulase | 1,4-β-glucan (cellulose) |
| GH10 | endo-1,4-β-xylanase | xylanase | xylan |
| GH13 | α-amylase | α-amylase | starch |
| GH16 | probable xyloglucan endotransglucosylase/hydrolase | xyloglucan endotransglucosylase/hydrolase | xyloglucan |
| GH17 | glucan endo-1,3-β-glucosidase | β-1,3-glucanase | 1,3-β-glucan |
| GH18 | chitinase; hevamine | Chitinase | chitin |
| GH19 | endochitinase | Chitinase | chitin |
| GH20 | β-hexosaminidase | β-hexosaminidase | a broad substrate specificity |
| GH27 | α-galactosidase | α-galactosidase (melibiase) | galactomannan |
| GH28 | polygalacturonase | polygalacturonase | homogalacturonan |
| GH29 | α-L-fucosidase 1 | α-L-fucosidase | xyloglucan |
| GH31 | α-xylosidase | α-xylosidase | xyloglucan |
| GH32 | β-fructofuranosidase, insoluble isoenzyme CWINV1 | β-fructofuranosidase | sucrose, 1-kestose |
| GH35 | β-galactosidase | galactosidase | galactans |
| GH37 | trehalase | trehalase | trehalose |
| GH38 | α-mannosidase | α-mannosidase | glycoproteins |
| GH51 | α-L-arabinofuranosidase | α-L-arabinofuranosidase | arabinoxylan, xylan, arabinan |
| GH65 | α-L-fucosidase 2 | α-L-fucosidase | xyloglucan |
| GH79 | heparanase-like | glucuronidase | Arabinogalactan-protein (AGP) |
| GH127 | DUF1680 domain protein | β-L-arabinofuranosidase | β-1,2-linked arabinofuranose disaccharide |
| Scheller HV, Ulvskov P (2010) Hemicelluloses. Ann Rev Plant Biol 61:263-289.  Caffall KH, Mohnen D (2009) The structure, function, and biosynthesis of plant cell wall pectic polysaccharides. Carbohyd Res 344:1879-1900. | | | |
